# Highlights on selected microscopy techniques to study zebrafish developmental biology

**DOI:** 10.1186/s42826-020-00044-2

**Published:** 2020-04-22

**Authors:** Ahmed Abu-Siniyeh, Walid Al-Zyoud

**Affiliations:** 1 Clinical Laboratory Sciences Department, College of Applied Medical Science, Taif University, Taif, Kingdom of Saudi Arabia; 2 Department of Biomedical Engineering, School of Applied Medical Sciences, German Jordanian University, Amman, Jordan

**Keywords:** Zebrafish, Development, Animal model, Two-Photon Microscopy, Second Harmonic Generation, Light-Sheet Microscopy

## Abstract

Bio-imaging is a tedious task when it concerns exploring cell functions, developmental mechanisms, and other vital processes in vivo. Single-cell resolution is challenging due to different issues such as sample size, the scattering of intact and opaque tissue, pigmentation in untreated animals, the movement of living organs, and maintaining the sample under physiological conditions. These factors might lead researchers to implement microscopy techniques with a suitable animal model to mimic the nature of the living cells.

Zebrafish acquired its prestigious reputation in the biomedical research field due to its transparency under advanced microscopes. Therefore, various microscopy techniques, including Multi-Photon, Light-Sheet Microscopy, and Second Harmonic Generation, simplify the discovery of different types of internal functions in zebrafish. In this review, we briefly discuss three recent microscopy techniques that are being utilized because they are non-invasive in investigating developmental events in zebrafish embryo and larvae.

## Introduction

Imaging a live organism is not easily accessible because matching a suitable animal model with a proper microscopy technique is challenging. In this context, different advanced microscopy approaches on different animal models were used either to explore the etiology of diseases or to explore the feasibility of discovering new drugs. However, some of these techniques suffer severe limitations.

The most advantageous features of zebrafish are its transparency, its amenability to optical imaging, in addition to the ease of generation of transgenic lines. These features lead zebrafish to be an excellent animal model for addressing questions for in vivo studies in vertebrates. Different applications were implemented for this purpose, especially advanced microscopy techniques such as Fluorescence Correlation Spectroscopy (FCS), Multi-Photon Microscopy (MPM), and Light-Sheet Microscopy (LSM) [1–3]. Reaching capabilities in elucidating and understanding pathophysiological events is a substantial step to reveal the anatomical and pathophysiological changes in vivo.

Here, we briefly discuss the engagement of zebrafish in three models of advanced microscopy techniques: Two-Photon Microscopy (TPM), Second Harmonic Generation, (SHG), and Light-Sheet Microscopy (LSM). Different reasons were behind selecting these techniques include non-invasiveness, the high resolution in imaging intact biological samples with deep penetration, and success in imaging live zebrafish embryos and larvae without affecting their viability or behavior.

## Zebrafish and microscopy

Transparency of zebrafish is a dominant feature; therefore, many recent studies implemented zebrafish with advanced microscopy to explore significant events related to vertebrate development, functional genomics, organ functions, behavior, toxicology, and drug discovery [4–6]. Live zebrafish embryos were employed in glycans imaging by treating zebrafish with an unnatural sugar to label their cell-surface glycans with azides metabolically. A significant increase in de novo glycan biosynthesis was recorded in different organs during development. The multicolor detection helped in conducting a spatiotemporal investigation of glycan trafficking and expression that cannot be achieved by conventional microscopy [7]. Apoptotic embryonic cells of zebrafish were visualized in vivo*,* and scientists performed successful labeling for apoptotic cells by genetically encoded fluorescent reporter protein. As an exceptional animal model, the authors described the patterns of apoptosis and the neuronal cell death at a single-cell resolution [8].

Later, apoptosis was investigated in live zebrafish larvae through implementing a combination of Fluorescence Lifetime Imaging (FLIM) with Optical Projection Tomography (OPT). In this work, the application of FLIM OPT was a pioneer in monitoring apoptosis over time in vivo with genetically expressed Förster Resonant Energy Transfer (FRET) biosensors [9].

Zhao and colleagues utilized probes in live zebrafish embryos with a high spatial resolution to verify a functional imaging method through multiple (FRET). They showed a possibility to measure the levels of Ca^2+^ and cAMP at low resolution by expressing dual FRET sensors. The FRET was rapid, robust, and compatible with a varied range of sensors for functional microscopy in vivo. Therefore, it was proposed that FRET would facilitate the imaging of complex cellular systems in whole live organisms [10]. A new ratiometric two-photon fluorescent probe (RN3) was recently synthesized to detect Palladium 2^+^ (Pd2^+^) in living zebrafish larvae by FRET, with a significant advantage in monitoring Pd2^+^ due to that RN3 possessed excellent biocompatibility and low cell cytotoxicity [11].

## Zebrafish by Two-Photon Microscopy

**Fig. 1:**
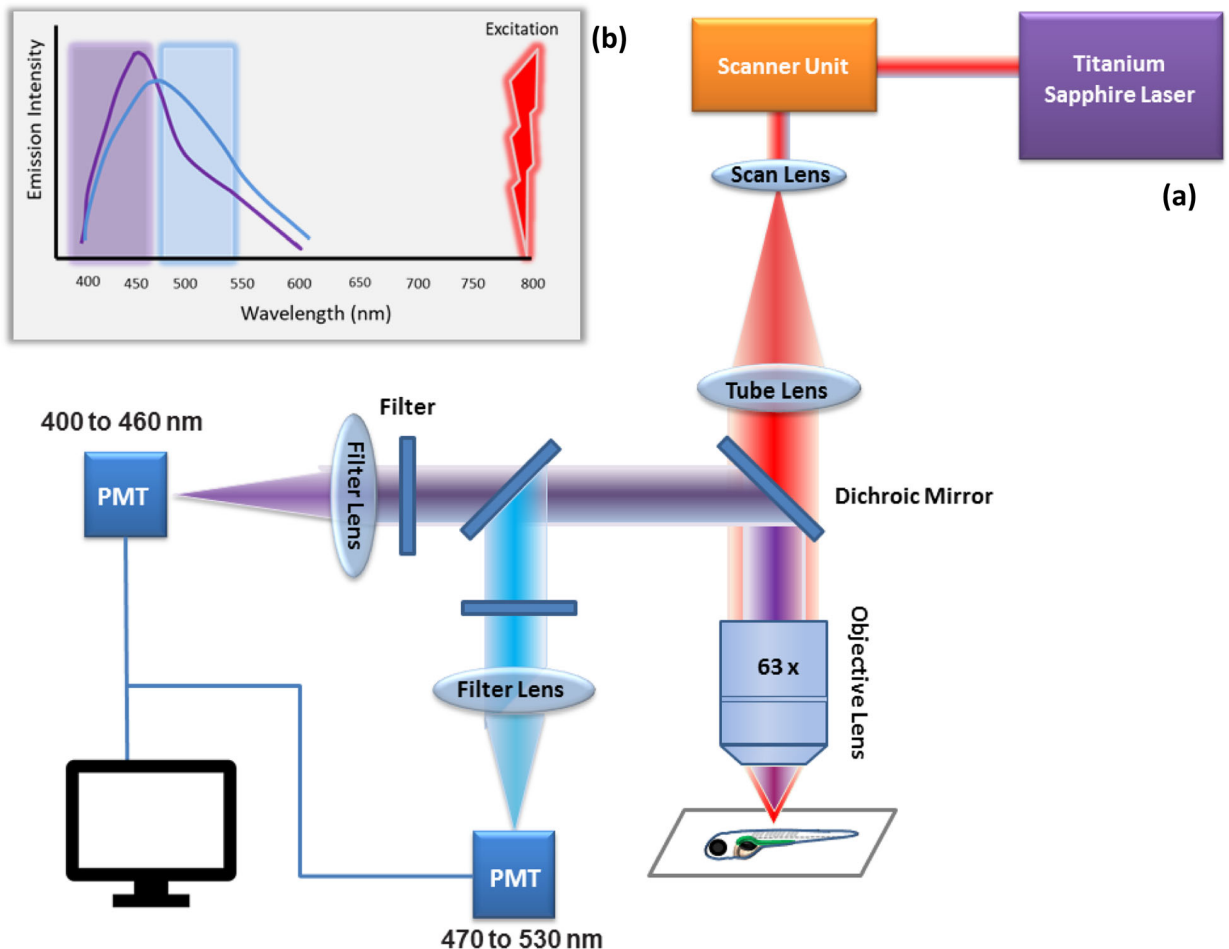
Schematic illustration of (**a**) Principle of imaging membrane order phases in zebrafish larvae by multiphoton with Laurdan dye. Titanium-Sapphire laser produces 800 nm wavelength, and the laser beam passes through a scanner unite, scan lens (SL), tube lens (TL) all focused on the zebrafish with × 63 1.4 numerical aperture (NA) of the water-immersion lens. The scattered signals from the sample focused on two photomultiplier tube detectors (PMT) with two wavelengths; 400–460 nm (for order phase) and 470–530 nm (for disorder phase) for PMT1 and PMT2, respectively. **b** Laurdan Fluorescence characteristics. The Laurdan dye is excited at 800 nm and its emission wavelength peaks at ~ 450 nm (violet) when existing in the ordered phase, and ~ 500 nm in the disordered phase (blue). The violet and blue boxes represent the acquisition channels conducted in the 400–460 nm and 470–530 nm wavelength bands, respectively

The new high-resolution and non-invasive imaging techniques on transparent zebrafish embryos enabled scientists to visualize morphogenetic activities during the early development of zebrafish and understand the cell membrane and its organization in a whole animal model [1, 12]. Many fluorescent lipid analogs and probes have been effectively implemented in zebrafish [13–15]. For example, new nonlinear optical techniques, included label-free visualization of lipids, have released new opportunities using zebrafish in lipid studies [16]. The first membrane image of lipid order in a whole living organism was reported in 2010. Laurdan and Two-Photon Microscopy were used to take three-dimensional images of embryonic cell membranes in zebrafish, where a high lipid order in the apical surfaces of polarized epithelial cells was observed [12] (Fig. 1).

Calcium imaging in the zebrafish in vivo included a two-photon microscope, as reported by Renninger and Orger 2013. Genetically encoded calcium indicators have successfully unlocked the constrains in recording neural population activity in zebrafish. Two-photon Microscopy enabled imaging the whole brain with single-cell resolution, generating interesting brain-wide functional maps in zebrafish [17].

**Fig. 2:**
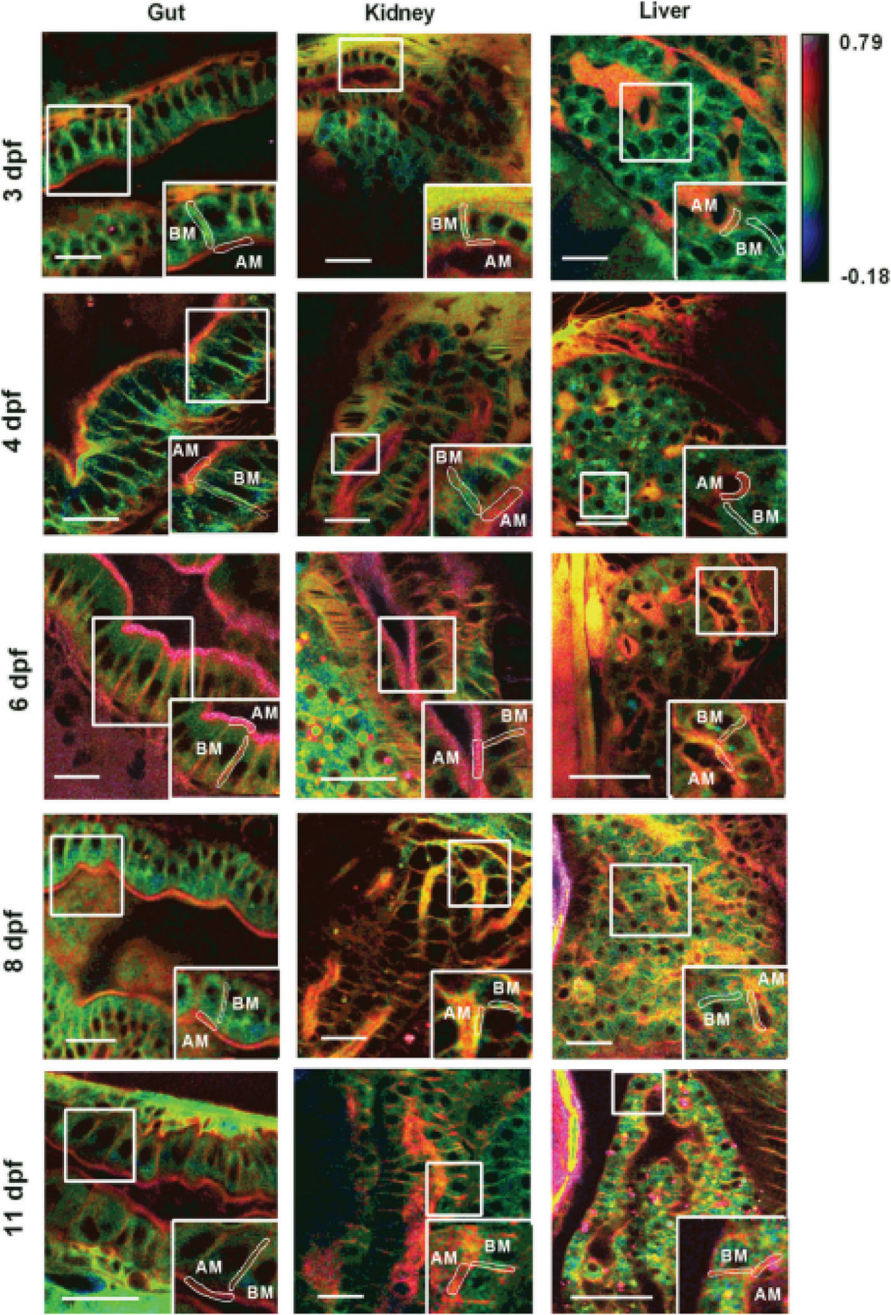
Acquired images for zebrafish tissues (gut, kidney, and liver) during different developmental stages, from 3 dpf to 11 dpf by using a Two-Photon microscope in conjugation with Laurdan dye. Scale bar = 20 μm. Adapted with permission from ref. [18]. Copyright 2016 John Wiley and Sons Periodicals. *days post-fertilization

By using deep Two-Photon Microscopy in zebrafish, microdomains in polarized epithelial cells were visualized by Laurdan dye, where the membrane order was quantified in the gut, kidney, and liver ductal epithelia during different developmental stages, from 3 to 11 dpf (days post-fertilization). It was found that the membrane order of polarized epithelial cells of the three types of tissues mentioned above is significantly changing during development with high membrane orders acquired at 6 dpf stage (Fig. 2). This observation emphasized the critical role of lipid rafts in cell functions and the organogenesis in zebrafish during early developmental stages [18].

## Drawbacks of Two-Photon Microscopy

Despite many advantages of Two-Photon Microscopy, there are also some drawbacks to such a technique. For example, photobleaching and photodamage are the most common artifacts at the focal volume, where photochemical interactions occur. Due to the high light exposure on the sample, it may induce unfavorable heat; this may lead to direct damage and considerable morphological changes to the sample, especially in pigmented tissue [19]. But this kind of damage might be reduced by exposing the sample to shorter laser pulses [20]. Non-linear damage may also occur by Two-Photon Microscopy, which are significant contributors to cell phototoxicity [21, 22]. Different approaches and modifications could be implemented to reduce phototoxicity and photodamage that might occur during live fluorescence microscopy, such as limiting sample illumination to a focal plane. Applying such a strategy allowing more consistent and reproducible image data acquisition in high resolution with wide-range periods of observation. In summary, limiting Two-Photon Microscopy drawbacks depends on well-controlled and well-designed experiments in terms of light exposure power and time [23].

## Zebrafish by Second Harmonic Generation

**Fig. 3:**
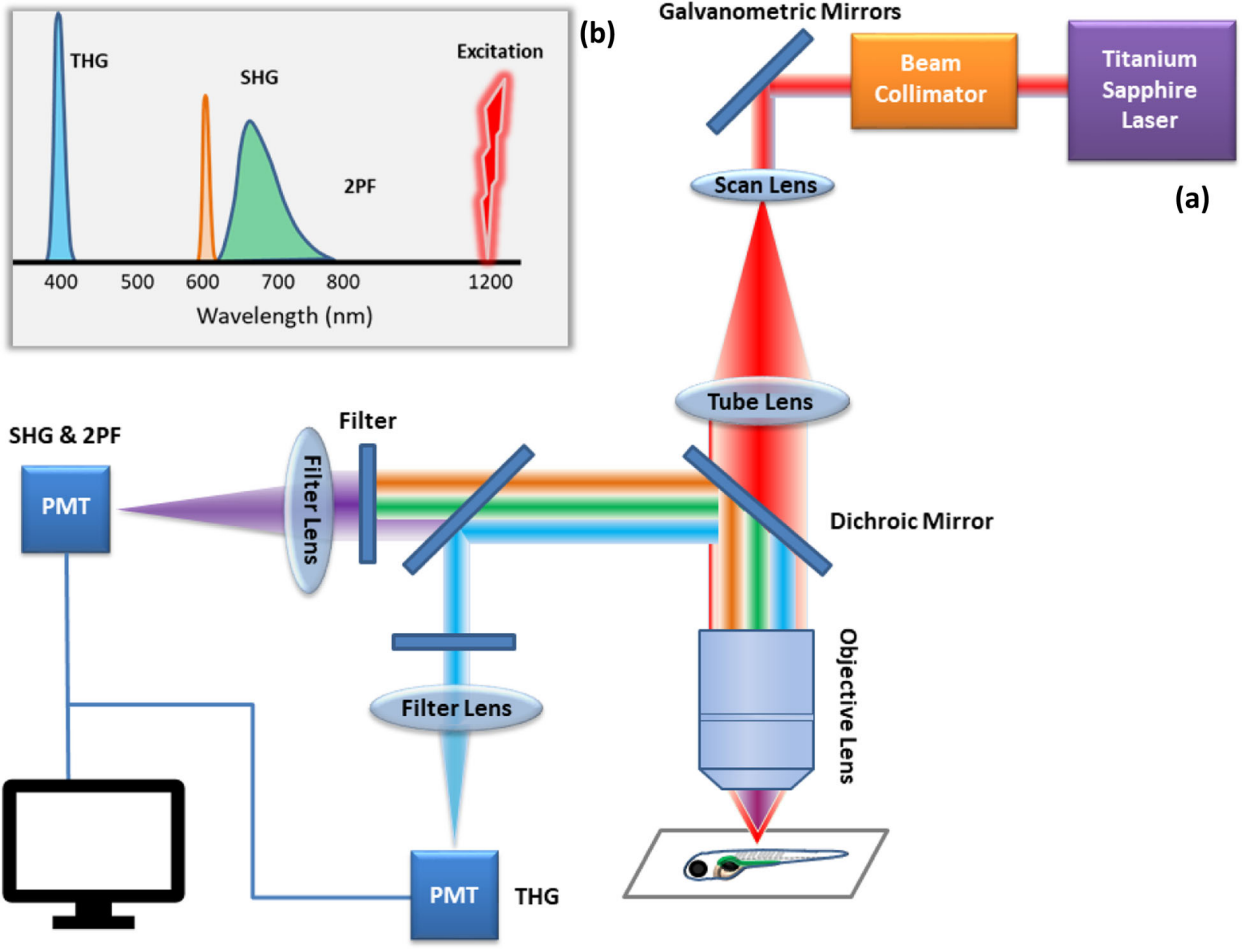
**a** A presentative diagram of imaging zebrafish embryo by Second Harmonic Generation Microscopy (SHG) and Third Harmonic Generation (THG): A titanium-sapphire laser generates pulses of 200 fs at 1200 nm. The laser beam passes on galvanometric mirrors (GM) to reach the sample through the objective lens. A dichroic mirror reflects the scattered signals from the sample, and the other dichroic mirror separates them into two channels (THG) and (SHG/2PF). The signals focused on the photomultiplier tube detectors (PMT), one for THG, and the other for SHG and 2PF. **b** A scheme of the light spectrum in SHG and THG microscopy. The excitation wavelength is 1200 nm, while the emission spectra of THG at 400 nm, SHG at 600 nm, and Two-Photon 2PF from 600 to 800 nm with a peak at ~ 650 nm generated by the sample

A higher harmonic generation technique, which includes the Second and Third Harmonic Generation, is noninvasive imaging utilized in zebrafish with a precise penetration, in the millimeter range, and sub-micron three-dimensional resolution (Fig. 3).

**Fig. 4:**
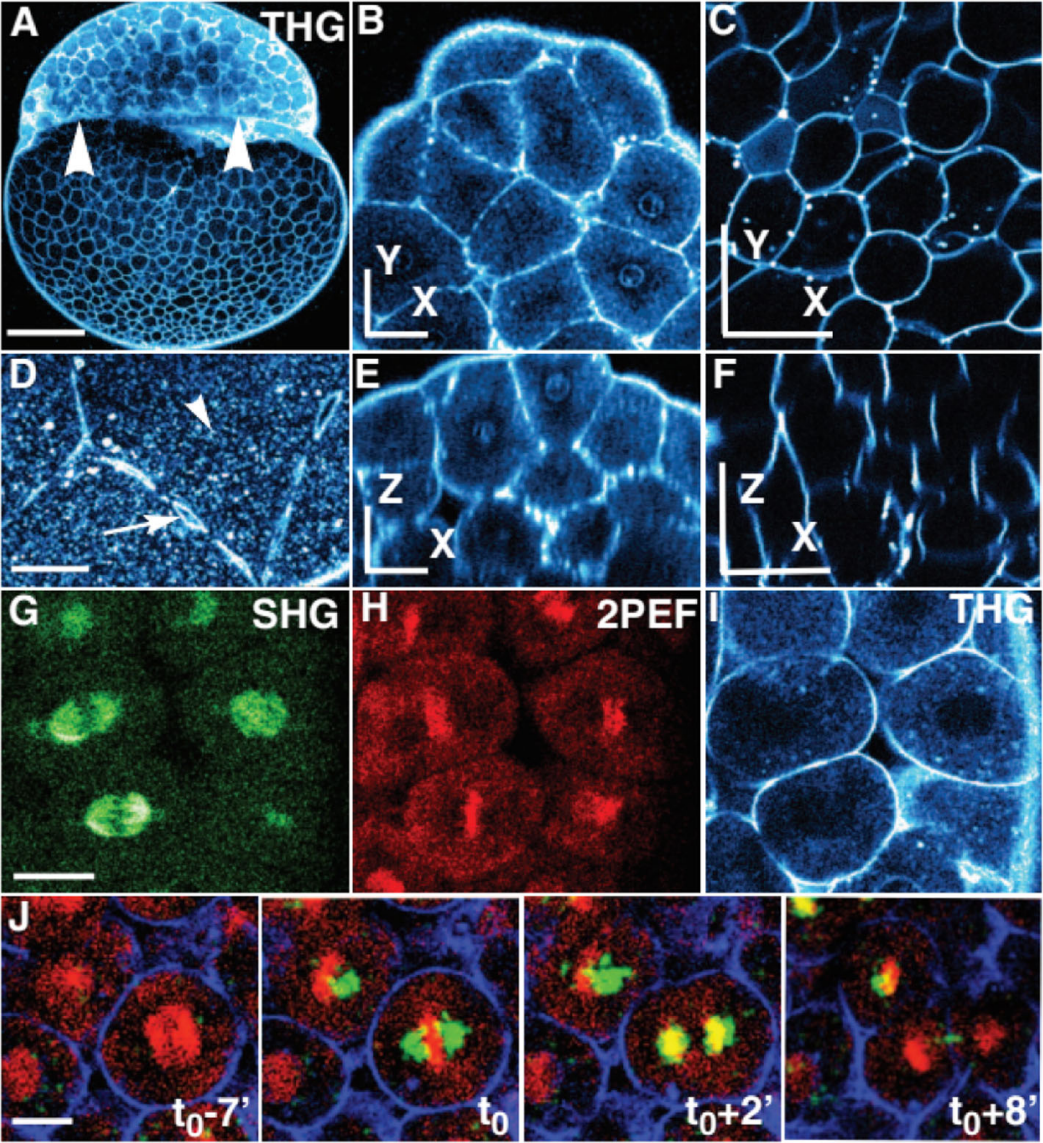
Presentative images of successful in toto imaging by using a combination of THG and SHG in addition to 2PEF for imaging unlabeled zebrafish embryo during the cleavage period. Adapted with permission from ref. [25]. Copyright 2010; The American Association for the Advancement of Science (AAAS) periodicals

In this technique, complex in vivo developmental processes in zebrafish embryos were successfully detected without any pretreatment within less than 1-mm-thick. Even with high illumination and long-term observations, no visible damage was detected, and no adverse effect was found on embryos until the larval stage. The three-dimensional resolution enabled the researchers to acquire images of cellular processes occurring inside embryos and larvae. This effective technique provided a glance at the dynamics of cytological construction during embryogenesis, which added valuable findings to the research of developmental biology [24, 25]. For example, cell behavior during zebrafish embryo cleavage stages was successfully reconstructed in a three-dimensional (3D) imaging by combining SHG with THG in imaging zebrafish embryos without any labeling [26] (Fig. 4).

Second Harmonic Generation (SHG) was employed to study the organization of collagen during the fin wound healing in zebrafish. The outcome of this work proposed the importance of collagen fibers during fin re-growth [27]. In a similar study, collagen reorganization during development and after wound healing was investigated in zebrafish larvae by Two-Photon, SHG imaging of fluorescently labeled transgenic macrophages. This technique allowed a non-invasive assessment of collagen fibers during development, wound healing, macrophage response, and potential interactions between macrophages and collagen fibers [28].

The transparency, genetic liability, and availability of fluorescently tagged immune cells of zebrafish with using the SHG imaging provided new perceptions to tissue regeneration in wound healing in a live animal.

## Drawbacks of Second Harmonic Generation

Despite the effectiveness of SHG microscopy in imaging biological tissue, it still suffers from some limitations. One of these limitations is the restricted penetration depths (100–300 μm with laser excitation in the 800–1000 nm range). Consequently, this might make SHG microscopy inappropriate for some biological applications, mainly when the region of interest is deeply located within the tissue or organ; thus, it is unreachable by SHG. The biggest issue in SHG microscopy is that it can image only a few structural proteins or harmonophores such as collagen types I & III, actomyosin complexes, cholesterol crystals (ChC), centrosomes and mitotic spindles. Unfortunately, to date, scientists could not find a method that can discriminate between fibrillar collagen types, which could enhance wound repair and regeneration biology [29]. However, these limitations might be controlled by finding suitable imaging solutions.

## Zebrafish by Light-Sheet Microscopy

**Fig. 5:**
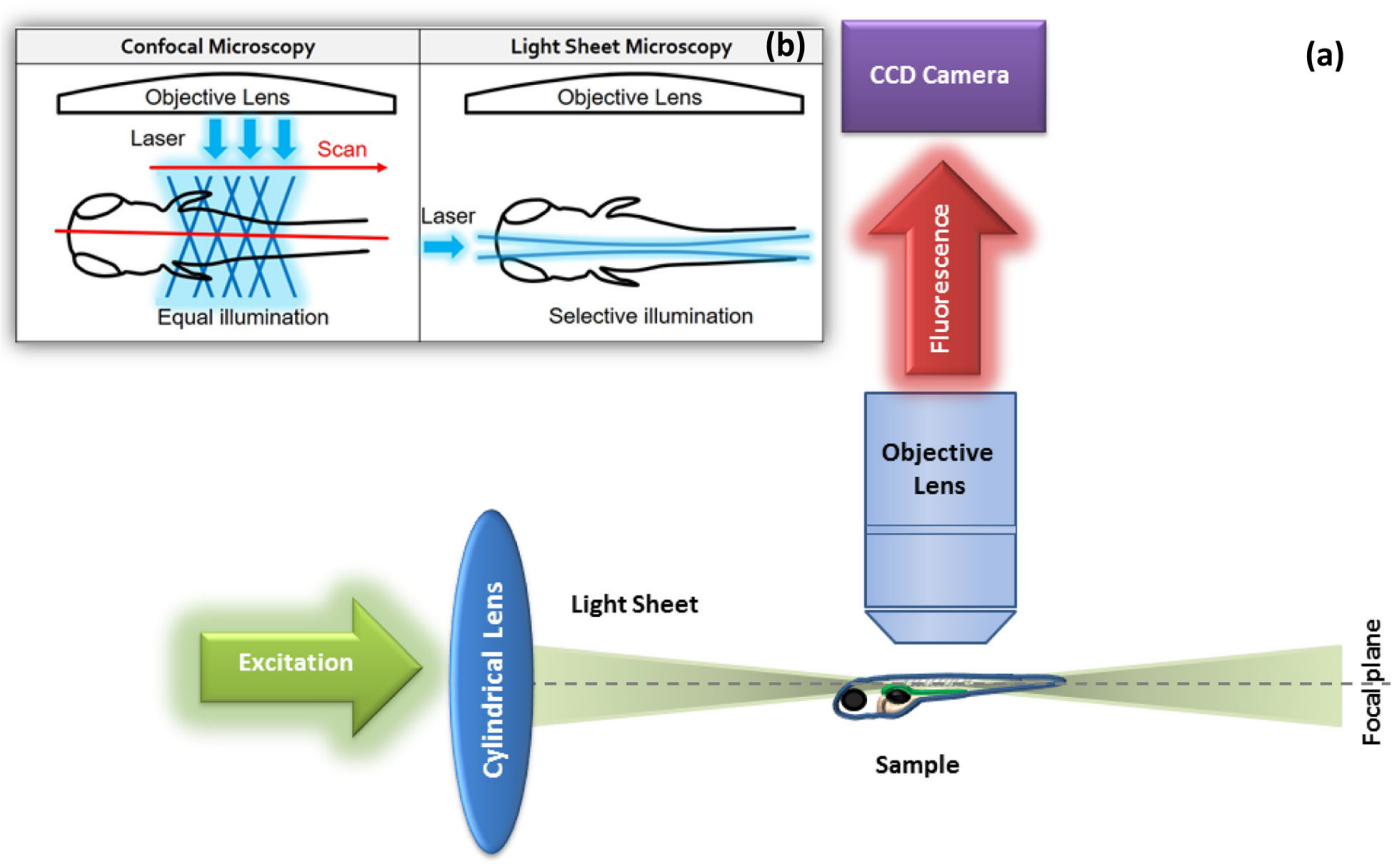
Schematic diagram of (**a**) LSM principle in imaging zebrafish larvae through illumination and detection at a right angle. Illumination (excitation) focused in one direction through a cylindrical lens in which a thin section of the sample is illuminated vertically to the orientation of observation. Objective lens used for fluorescence detection perpendicularly to the sample. **b** LSM uses a planar illumination of the focal plane from the side (selective illumination) instead of a point illumination as in confocal microscopy (equal illumination), which enables LSM to capture images at a faster speed, reducing photodamage and offering optical sectioning compared to confocal microscopy

By exploiting the optical features of zebrafish, Light-Sheet Microscopy (LSM) became a preferred technique in imaging embryonic development. LSM owns various strengths such as deep optical sectioning, high frame rates, faint excitation intensities, minimal phototoxicity, moderate mounting techniques, rapid imaging with an efficient signal to noise ratio, and imaging specimens from different angles for a prolonged time [30, 31] (Fig. 5). In 2008, a digital scanned laser Light-Sheet Fluorescence Microscopy was established to report nuclear movement and localization of wild type and mutant zebrafish embryos. They were able to measure the embryonic body axis based on morphodynamic symmetry. They also created a germ layer model that demonstrated that the mesendoderm development was formed around 33% of the embryonic cells during a given phase of cell division [32]. Later, the zebrafish brain/inner ear region was successfully imaged by using improved Thin-Sheet Laser Imaging Microscope (TSLIM) at a comparable resolution of wide-field fluorescence microscopy succeeded in delivering an even section of illumination and decreasing penetration artifacts that the Light-Sheet Microscopy suffered from [33].

**Fig. 6:**
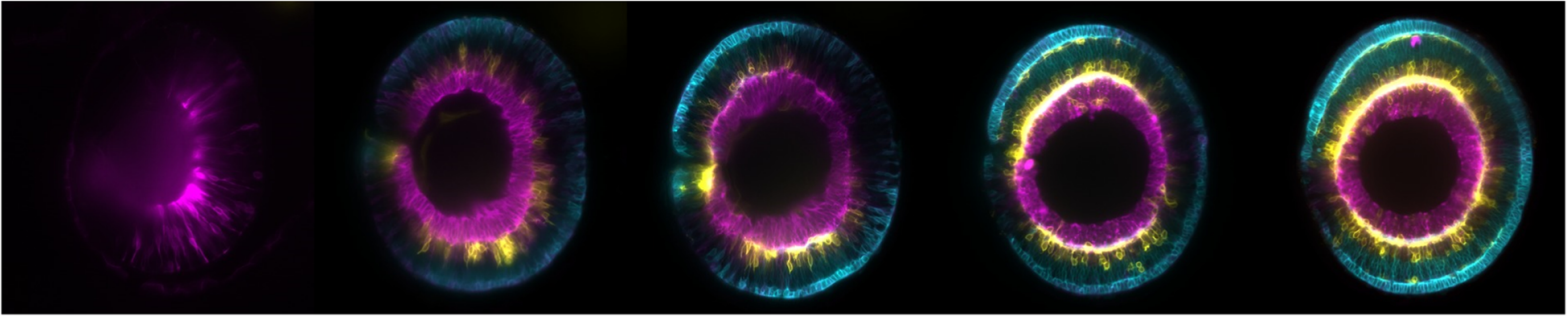
Different developmental stages of the retina of zebrafish embryo acquired by Light Sheet Microscopy (LMS) from 1.5 dpf to 3.5 dpf*. The retinal ganglion cells are shown in (magenta), the amacrine and horizontal cells are shown in (yellow), and the photoreceptors and bipolar cells are shown in (cyan). The image was created on 30 November 2015 by IchaJaroslav in the Norden lab at the Max Planck Institute of Molecular Cell Biology and Genetics (MPI-CBG) in Dresden, Germany, this file is licensed under the Creative Commons Attribution-Share Alike 4.0 International license. *days post-fertilization

Genetically encoded calcium indicator GCaMP5G was applied by Light-Sheet Microscopy to study the in vivo imaging of more than 80% of neurons in zebrafish larval brains. In a study, two populations of neurons with correlated activity patterns were successfully identified [33]. Also, Light Sheet Fluorescence Microscopy (LSFM) was efficient in studying eye morphogenesis in zebrafish emphasizes that LSFM could be better than conventional confocal imaging for this purpose [30] (Figs. 5b, 6).

LSFM requires taking a large *z* stack and a large field of view to display the main morphogenetic relocations followed the changeover from optic vesicle to the optical cup stage [34]. Wild type and mutant zebrafish were compared in vivo by Light-Sheet Microscopy to explore the activity of inhibitory neural networks during GABAergic signaling changing. Light-Sheet Microscopy succeeded in imaging the neural activity in two colors at 23 frames per second for over 10 min, which granted an opportunity to record the infrequent seizure crisis in mutant type, concluded that zebrafish might be used to mimic epilepsy in human [35]. Hence zebrafish became a recognized model in studying vascular development and disease in vivo [36].

Due to limitations in the optical range and scanning speed in confocal or Two-Photon Microscopy, it was challenging to image the whole zebrafish embryonic brains. Light-Sheet Fluorescence Microscopy (LSFM) was an efficient approach in imaging the entire head of live transgenic zebrafish embryos. By creating an inclusive 3D maps, researchers were able to image cranial neurons and blood vessels during zebrafish embryogenesis by LSFM. Blood cells circulating through the entire head, vagal, and cardiac vasculature at high resolution in a 3D were visualized, this came up with establishing a complete 4D atlas of embryogenesis and neural activity in zebrafish [37].

## Drawbacks of Light-Sheet Microscopy

Although LSFM is the only technique allowing volumetric imaging without exposing the sample to light outside the focal plane, LSFM frequently needs modifications in the experimental design and has illumination issues [30]. When high-speed acquisition needs to be achieved during LSFM imaging, high peak power is required for the line-by-line illumination applied to produce a scanned light sheet. High peak power can impose the intrinsic Reactive Oxygen Species (ROS) scavenging mechanisms of living organisms, which induce photodamage in the biological sample. Light-Sheet Microscopy needs a large amount of memory storage, which requires users to have impressive data storage pipelines. But this issue can be resolved by establishing a pipeline that doubles up the acquisition speed and reduces storage requirements [38]. Extra optics are required to produce the light sheet that may introduce steric constraints to the imaging system and sample mounting, which can be resolved by inducing modifications in the illumination architecture [39].

The principle, advantages, disadvantages, and the applications of the Two-Photon, Second Harmonic Generation, and Light-Sheet Microscopy are tabulated in (Table 1).

**Table 1:** Summary of three microscopy techniques used in zebrafish studies

Method	Principle	Advantages	Disadvantages	Applications on zebrafish	References
Two-Photon Microscopy	• Based on nonlinear optical processes. • Image fluorescent dyes or endogenous molecules. • Near-infrared light is used instead of visible. • Two-photon excited fluorescence is based on the simultaneous absorption of two lower-energy photons.	• Suitable for imaging optically thick specimens. • Less scattering and absorption in biological tissue, permitting for deeper penetration. • No pinhole aperture and minimizes signal loss. • Photobleaching of fluorescent molecules outside the focus is almost abolished. • High resolution in imaging intact biological samples without spatial filtering.	• Photobleaching within the focal volume, with laser power levels typically used in biological imaging. • Induce considerable photodamage at the focal volume where photochemical interactions occur.	1. Study morphogenetic movements during early zebrafish embryonic development 2. Measure membrane order in tissues of zebrafish larvae. 3. Neural Population Activity in Zebrafish 4. Studying membrane order polarity proteins in the gut, kidney, and liver during vertebrate organogenesis.	1. Carvalho & Heisenberg 2009 [1] 2. Owen et al. 2010 [12] 3. Renninger & Orger 2013 [17] 4. Abu-Siniyeh et al. 2016 [18]
Second Harmonic Generation	• Used to image non-centrosymmetric structures such as collagen fibers and • A nonlinear optical process where two photons are converted to a single photon without losing any energy.	• Visualizes the tissue structure directly because the contrast is produced from endogenous species. • Significantly reduced photobleaching and phototoxicity compared to fluorescence methods. • It can reach high-resolution imaging to several hundred microns depths.	• Limited penetration depths 100–300 μm with laser excitation in the 800–1000 nm range to increase image resolution. • Micrometer depths are often inadequate for in vivo applications.	1. Collagen organization in zebrafish during wound healing. 2. Gene expression observation in zebrafish embryo nerve systems.	1. LeBert et al. 2015 [27] 1. LeBert et al. 2016 [28] 2. Hsieh et al. 2008 [40]
Light-Sheet Microscopy	• The defining feature of LSM is the planar illumination of the focal plane from the side. • Only a thin section of the sample is illuminated at any given time.	• Rapid imaging with high frame rates • High signal-to-noise ratios. • Minimum rates of photo-bleaching and toxicity. • Three-dimensional imaging of live samples. • Minimized photodamage. • Deep optical sectioning. • Faint excitation intensities. • Moderate mounting techniques.	• Extra optics are required to produce the light sheet. • Adding the extra lens introduces steric constraints to the imaging system and sample mounting.	1. Image zebrafish eye development 2. Imaging a seizure model in zebrafish 3. Zebrafish vascular development 4. Brain functional imaging 5. 3D imaging of cranial neurons and vasculature during zebrafish embryogenesis.	1. Icha et al. 2016 [34] 2. Kner et al. 2018 [35] 3. Kugler et al. 2018 [36] 4. Misha et al. 2013 [41] 5. Ok Kyu Park et al. 2015 [42]

## Conclusion

Despite arguments and discrepancies about the visibility of using zebrafish as a research model, different zebrafish studies have been performed without changing its viability or behavior. Therefore, it is proven that zebrafish is a promising model in the microscopy field with a variety of applications due to its robust nature, its optical amenability, and genetic traceability.

Two-Photon Microscopy is a promising approach in studying different topics in live zebrafish. Photobleaching within the focal volume is the main weak point of Two-Photon Microscopy when a proper laser intensity is used; the photodamage to the zebrafish organs during image acquisition is nearly absent.

Two-Photon also showed a virtuous capability in zebrafish embryo imaging; hence, it is considered a powerful tool for addressing current issues in systems biology and high-content experimental investigation of embryonic development.

The Second Harmonic Generation (SHG) has many advantages. It is suitable to image intact and living tissues without labeling. Besides, its near-infrared excitation is nonlinear, which enables optical sectioning with minimal autofluorescence background and minimal photochemical damage of biological samples. SHG involves no absorption because it is a non-resonant process. The constraint of weak signal intensity of harmonic generation has been fixed by increasing the repetition rate of the excitation light.

SHG imaging is a promising tool for studying structural proteins in-vivo*,* mainly collagen and the muscle sarcomeres of zebrafish during its early development. It became more efficient in zebrafish when it used with genetically encoded fluorescent tags. There is a potential advantage of combing SHG with THG as this could identify gene expression levels in the embryonic nervous system in zebrafish in addition to recording and imaging the performance of the cell during the developmental stages of the zebrafish embryo (Fig. 4).

Light-Sheet Microscopy (LSFM) enables rapid imaging, high signal-to-noise ratios, and minimum rates of photobleaching and toxicity, three-dimensional imaging of live samples. These capabilities allowed scientists to quantify cellular dynamics in entire embryos throughout their development. LSFM owns an advanced ability, such as 4D in vivo imaging to reveal volumetric cellular structures in tissues during development. The LSFM has been combined with Structured Illumination (SI) to eliminate scattered and out-of-focus light. This powerful combination enhanced the resolution and contrast in the vertical and axial directions during imaging neural activity at the zebrafish larval stage.

In summary, each of these three advanced microscopy techniques owns its advantages and disadvantages when it was implemented in vivo studies. In the future, we recommend for further improvement by combining two different modalities for addressing more effective visualization. A successful model was an integration of one and Two-Photon scanned oblique plane illumination microscopy that provided light-sheet scanning based rapid volumetric imaging ability at subcellular resolution. High-resolution nonlinear optical microscopy with zebrafish as an animal model can revoke many challenges that limit detecting changes at the tissue structure during pathoetiologies.

## Data Availability

The dataset supporting the conclusions of this article is included within the article and its additional file.

## Abbreviations

ChC: Cholesterol Crystals
dpf: days post fertilization
FCS: Fluorescence Correlation Spectroscopy
FLIM: Fluorescence Lifetime Imaging
FRET: Förster Resonant Energy Transfer
GCaMP5G: Genetically encoded calcium indicator
GCaMP6: Green fluorescent calcium biosensor
GFP: Green Fluorescent Protein
LSFM: Light Sheet Fluorescence Microscopy
LSM: Light-Sheet Microscopy
MPM: Multi-Photon Microscopy
OPT: Optical Projection Tomography
Pd2+: Palladium 2^+^
ROS: Reactive Oxygen Species
SHG: Second Harmonic Generation
SI: Structured Illumination
TPM: Two-Photon Microscopy
TSLIM: Thin-Sheet Laser Imaging Microscope
